# Case report: Acute submandibular sialadenitis in poorly controlled diabetes mellitus patient fed twenty days by enteral tube

**DOI:** 10.1097/MD.0000000000034193

**Published:** 2023-07-07

**Authors:** Yuichiro Iwamoto, Takatoshi Anno, Katsumasa Koyama, Koichi Tomoda, Tomohiko Kimura, Hideaki Kaneto

**Affiliations:** a Department of Diabetes, Endocrinology and Metabolism, Kawasaki Medical School, Kurashiki, Japan; b Department of General Internal Medicine 1, Kawasaki Medical School, Okayama, Japan.

**Keywords:** acute submandibular glanditis, elderly patient, nasogastric tube feeding, parenteral nutrition, type 2 diabetes mellitus

## Abstract

Enteral tube feeding is an effective method of providing nutrients for patients who are unable to meet their nutritional requirements, and patients with parenteral nutrition are at an increased risk of infection. The submandibular gland is one of the major salivary glands and sialadenitis are often caused by obstruction of the salivary outflow tract.

**Patient concerns:**

A 91-year-old woman had parenteral nutrition with nasogastric tube feeding. Her history includes angina pectoris, myocardial infarction, type 2 diabetes mellitus (T2DM), heart failure, atrial fibrillation, sick sinus syndrome, and she recently had a pacemaker implanted. She was continued parenteral nutrition with nasogastric tube feeding for 20 days, and her fasting blood glucose ranged from 200 to 400 mg/dL. In the midst of poor glycemic control, she suddenly had high fever and elevated infection markers under poorly glycemic control.

**Diagnoses:**

She had neck swelling with a feeling of heat. We performed cervical computed tomography, and it revealed swelling of the bilateral submandibular glands and fluffing of surrounding tissue. She was diagnosed with acute submandibular glanditis.

**Interventions:**

We treated her with antibiotics therapy, extubation, daily massage of the submandibular gland and strict glycemic control.

**Outcomes:**

Her neck swelling disappeared about 11 days after such treatment.

**Lessons:**

We reported acute submandibular glanditis induced by nasogastric tube feeding under poorly controlled diabetes mellitus. We have to keep good oral hygiene and also pay attention to glycemic control in subjects under parenteral nutrition with tube feeding management.

## 1. Introduction

Enteral tube feeding is an effective method of providing nutrients for patients who are unable to meet their nutritional requirements. Oral environment in subjects with long-term tube feeding management frequently leads to various clinical problems. It is known that patients with parenteral nutrition are at increased risk of respiratory and intra-abdominal abscess infections.^[1]^ The submandibular gland is one of the salivary glands and some of sialadenitis are caused by obstruction of the salivary outflow tract, tumor, and after radiation therapy.^[2]^ In such cases, congested and bacteria-laden saliva easily flows back into the salivary glands. The obstruction can be due to various reasons such as a stone, thick mucus, or a tumor. When the outflow tract is blocked, saliva builds up in the gland, causing inflammation and pain. Other causes of sialadenitis may include viral or bacterial infections, autoimmune diseases, and certain medications. There is a case report of a neonate who developed aseptic sialadenitis during tube feeding but was improved with antimicrobial therapy.^[3]^ However, there have been few reports of sialadenitis during tube feeding in adults.

In this report, we show a subject who had acute submandibular glanditis caused by nasogastric tube feeding under poorly controlled diabetes mellitus. Her acute submandibular glanditis was improved with extubation of nasogastric tube and control of diabetes mellitus.

## 2. Case presentation

**Figure 1.**
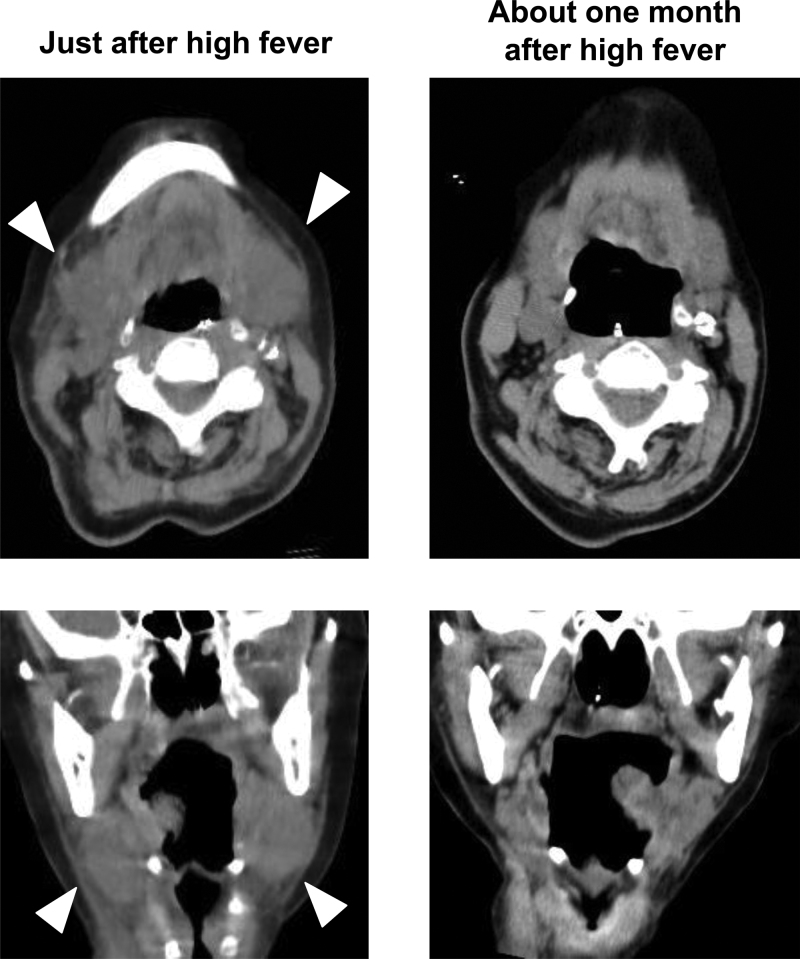
Cervical computed tomography (CT) after high fever in this subject receiving parenteral nutrition with nasogastric tube feeding for 22 days in our hospital. Cervical CT revealed swelling of the bilateral submandibular glands and fluffing of surrounding tissue (white arrowhead) (left panel). Cervical CT about 1 month after high fever showed that enlargement of submandibular gland was drastically improved receiving parenteral nutrition with nasogastric tube feeding for 57 days (right panel).

A 91-year-old woman repeated admission and discharge due to aspiration pneumonia and myocardial infarction. After she received pacemaker placement for sick sinus syndrome and aspiration pneumonia, she had parenteral nutrition with nasogastric tube feeding. She was taking 400 kcal of glucerna therapeutic nutrition shake at twice a day (breakfast and dinner) and 200 kcal of dysphagia diet at once a day (lunch). Her background history included repeated angina and myocardial infarction (at the age of 83, 84, 89, and 91) and performed percutaneous coronary intervention and coronary artery bypass grafting, type 2 diabetes mellitus (T2DM; at 83), heart failure, atrial fibrillation and sick sinus syndrome, and performed pacemaker placement (at 91). Her glycemic control was poor (her fasting plasma glucose levels were from 200 to 400 mg/dL) with insulin therapy (18 units/d of insulin aspart and 6 units/d of insulin degludec) after started with parenteral nutrition. Her medications included 30 mg/d of azosemide, 500 mg/d of acetazolamide, 25 mg/d of spironolactone, 1.25 mg/d of bisoprolol, 2.5 mg/d of enalapril, 30 mg/d of edoxaban, and 40 mg/d of isosorbide for hypertension, heart failure, and after coronary artery bypass grafting. We continued parenteral nutrition with nasogastric tube feeding for 20 days in our hospital, and suddenly she had high fever and elevated infection markers. At that time, her height and body weight were 150.0 cm and 40.3 kg/m^2^, respectively. Her vital signs were: temperature, 38.2 °C; blood pressure, 122/50 mm Hg; heart rate, 72 bpm; oxygen saturation, 96%. On clinical examination, she had neck swelling, which was bilateral, symmetric, elastic, and hard with a feeling of heat. The cervical lymph nodes were not enlarged and there was no tenderness. Adequate observation of the oral cavity was not possible due to difficulty opening the mouth associated with the cervical swelling. Table 1 shows laboratory data at that time. Her glycemic control was poor. Diabetes-associated data were as follows: plasma glucose, 335 mg/dL; hemoglobin A1c, 8.6%. Liver function and renal function were almost within normal range. Surprisingly, inflammation markers were markedly elevated compared with 3 days before: white blood cell, from 18,980 to 31,430/μL (neutrophil, from 80.0% to 91.0%); C-reactive protein, from 1.61 to 20.11 mg/dL; procalcitonin, from 0.05 to 1.44 ng/mL. We performed cervical, chest, and abdominal computed tomography (CT) for finding infection focuses, and it revealed swelling of the bilateral submandibular glands and fluffing of surrounding tissue (Fig. 1, left panel). There were no enlarged cervical lymph nodes or sialolithiasis. We did not detect another infectious focus including newly aspiration pneumonia. We discussed the possibility of acute submandibular glanditis caused by nasogastric tube feeding with otorhinolaryngologists, and finally we diagnosed her as acute submandibular glanditis.

**Table 1:** Laboratory data observed at fever up to 20 days after the start of nasogastric tube feeding.

Variable	Result	Reference range
Blood biochemistry		
Total protein (g/dL)	6.3	6.6–8.1
Albumin (g/dL)	2.1	4.1–5.1
Globulin (g/dL)	4.2	2.2–3.4
Total bilirubin (mg/dL)	0.4	0.4–1.5
AST (U/L)	30	13–30
ALT (U/L)	51	7–23
LDH (U/L)	198	124–222
ALP (U/L)	385	106–322
γ-GTP (U/L)	21	9–32
BUN (mg/dL)	24	8–20
Creatinine (mg/dL)	0.50	0.46–0.79
Cholinesterase (U/L)	121	201–421
Uric acid (mg/dL)	6.3	2.6–5.5
Sodium (mmol/L)	133	138–145
Potassium (mmol/L)	2.6	3.6–4.8
Chloride (mmol/L)	104	101–108
Peripheral blood		
White blood cells (/μL)	31,430	3300–8600
Neutrophil (%)	91.0	28.0–78.0
Red blood cells (×10^4^/μL)	304	386–492
Hemoglobin (g/dL)	10.1	11.6–14.8
Hematocrit (%)	28.8	35.1–44.4
Platelets (×10^4^/μL)	27.3	15.8–34.8
Infectious marker		
CRP (mg/dL)	20.11	<0.14
Procalcitonin (ng/mL)	1.44	0.00–0.05
Diabetes marker		
Plasma glucose (mg/dL)	335	
Hemoglobin A1c (%)	8.6	4.9–6.0
Urinary test		
Urinary pH	5.5	5.0–7.5
Urinary protein	1+	–
Urinary sugar	3+	–
Urinary ketone body	–	–
Urinary bilirubin	–	–
Urinary blood	–	–
Urinary pH	5.5	5.0–7.5

ALP = alkaline phosphatase, ALT = alanine aminotransferase, AST = aspartate aminotransferase, BUN = blood urea nitrogen, CRP = C-reactive protein, LDH = lactate dehydrogenase, γ-GTP = γ-glutamyl transpeptidase.

We started antibiotics therapy for acute submandibular glanditis (3 g/d of sulbactam/ampicillin). In addition, we performed extubation and daily massage of the submandibular gland to improve saliva secretion (20 minutes of massage with physician and several times of massage with nurse around the submandibular gland area every day) and oral environment with oral care with nurse.

Moreover, we performed strict glycemic control (her fasting plasma glucose levels were from 120 to 180 mg/dL) with insulin therapy (36 units/d of insulin aspart and 6 units/d of insulin degludec), although she was elderly patient. High fever was improved about 3 days after starting antibiotics therapy and submandibular gland massage, and her neck swelling disappeared about 11 days after such treatment. In addition, her infectious markers were drastically decreased (white blood cell, from 31,430 to 10,280/μL; C-reactive protein, from 20.11 to 1.50 mg/dL; procalcitonin from 1.44 to 0.02 ng/mL). Cervical CT showed that such enlargement of submandibular gland was improved (Fig. 1, right panel). Finally, she was transferred to another hospital for rehabilitation 38 days after admission.

## 3. Discussion and conclusions

Herein, we report a subject with acute submandibular glanditis caused by nasogastric tube feeding under poorly controlled diabetes mellitus. Acute submandibular glanditis could be caused by the reflux of bacteria-laden saliva into the submandibular gland ducts. Therefore, the bacterial reflux into the salivary glands causes the rapid onset of pain and swelling in the salivary gland area, and palpation reveals induration, edema, and tenderness. *Staphylococcus aureus* is the most common causative organism of bacterial sialadenitis in both adults and children.^[4]^ The risk factors of sialadenitis are dehydration, oral filth, dental caries, oral trauma, xerostomia, low nutrition, and diabetes mellitus.^[5]^ It has been reported that diabetics have decreased saliva secretion from their salivary glands.^[6]^ It is known that in patients under hyperglycemia condition^[7]^ or patients complicated with diabetic neuropathy,^[8]^ saliva secretion is reduced, leading to dry mouth, increased tooth decay, and salivary gland inflammation. Our patient probably developed acute submandibular adenitis due to a combination of factors, including poorly controlled diabetes mellitus and decreased swallowing frequency and saliva production due to tube feeding. Since diabetes mellitus is known to cause progressive inflammation in various tissues, we assume that the presence of diabetes mellitus was closely related to the development of acute submandibular adenitis due to nasogastric tube feeding in this case.

There are some reports about acute submandibular glanditis associated with anesthesia and intubation, so-called anesthesia mumps.^[9,10]^ Procedures that decrease the frequency of swallowing, such as tracheal intubation, stagnate saliva flow, resulting in bacterial sialadenitis.^[11]^ Sialadenitis in the elderly is most common in the parotid gland, but there have been several reports of post-intubation sialadenitis occurring in the submandibular gland. We assume that in the patient, swallowing frequency was decreased due to tube feeding and consequently salivary secretion was decreased, which finally led to the development of acute submandibular glanditis. Interestingly, we think it is very important to perform strict glycemic control in elderly patients with long-term tube feeding management for infectious disease control. T2DM subjects are immunocompromised host, especially under hyperglycemic conditions.^[12,13]^ Because sialadenitis develops against a multifactorial background, identifying a single cause is not easy, and careful evaluation of the general condition is important.

For treatment of acute submandibular glanditis, it is important to perform not only antibiotics therapy but also salivary gland massage, hydration, salivary stimulants, and glycemic control.^[14]^ Performing oral care, including salivary gland massage during tracheal intubation, improves salivary stagnation, especially in the elderly over 65 years of age.^[15]^ In general, penicillin-based antibiotics are used, but we must be careful about penicillin-resistant bacteria.^[16]^ Although the saliva culture test was negative in this case, it was difficult to collect the specimen because of the decreased salivary secretion at the time of antimicrobial administration. Therefore, we cannot exclude the possibility that the specimen was negative just because it was submitted after salivary gland massage. We empirically chose to treat this patient with penicillin and cephem because of the high risk of bacterial resistance, especially in patients on tube feedings or hospitalized for a long period of time. If sialadenitis is suspected, ultrasound and magnetic resonance imaging, as well as CT, are useful for diagnosis.^[2]^ It is also important to confirm the findings of the salivary duct opening in the oral cavity. Although the inflammation in this case improved quickly with conservative treatment, it is important to aggressively perform several imaging studies if abscess formation or refractory treatment is anticipated. Severe acute sialadenitis can also result in upper airway obstruction, requiring monitoring of vital signs and a decision to intubate the trachea if the situation warrants.^[11]^

Taken together, we should bear in mind that parenteral nutrition with long-term tube feeding management is one of risk factors of acute submandibular glanditis. In addition, it is likely that the risk is closely associated with glycemic control of T2DM, especially under poorly controlled conditions. Therefore, we have to pay attention to glycemic control in subjects under parenteral nutrition with tube feeding management.

## Author contributions

**Conceptualization:** Yuichiro Iwamoto.

**Writing – original draft:** Yuichiro Iwamoto.

**Writing – review & editing:** Takatoshi Anno, Katsumasa Koyama, Koichi Tomoda, Tomohiko Kimura, Hideaki Kaneto.
